# Characterization of the Key Aroma Compounds in Marselan Wine by Gas Chromatography-Olfactometry, Quantitative Measurements, Aroma Recombination, and Omission Tests

**DOI:** 10.3390/molecules24162978

**Published:** 2019-08-16

**Authors:** Jiaheng Lyu, Yue Ma, Yan Xu, Yao Nie, Ke Tang

**Affiliations:** 1 Key Laboratory of Industrial Biotechnology of Ministry of Education, Jiangnan University, 1800 Lihu Ave, Wuxi 214122, Jiangsu, China; 2 State Key Laboratory of Food Science and Technology, Jiangnan University, 1800 Lihu Ave, Wuxi 214122, Jiangsu, China

**Keywords:** Marselan wine, GC-O, AEDA, GC × GC-TOF-MS, OAVs, aroma reconstitution, omission tests

## Abstract

Key odorants of red wine made from the hybrid grapes of Marselan (*Vitis vinifera* L.) were isolated by solid-phase extraction (SPE) and explored by gas chromatography-olfactometry (GC-O) analysis. Application of aroma extract dilution analysis (AEDA) revealed 43 odor-active compounds, and 31 odorants among them were detected with flavor dilution (FD) factors ranging from 9 to 2187. Comprehensive two-dimensional gas chromatography and time-of-flight mass spectrometry (GC × GC-TOF-MS) were exploited to quantitate the aroma-active compounds with FD ≥9. The identification indicated *β*-damascenone as having the highest FD factors, followed by eugenol, 2,3-butanedione, citronellol, 4-hydroxy-2,5-dimethyl-3(2*H*)-furanone, phenethyl acetate, guaiacol, and 2-methoxy-4-vinylphenol. A total of 21 compounds were found to have odor activity values (OAVs) >1.0. Aroma reconstitution validation experiments showed a good similarity of blackberry, green pepper, honey, raspberry, caramel, smoky, and cinnamon aroma attributes between the original Marselan wine and the reconstructed wine. In addition, omission tests were carried out to further determine the contribution of odorants to the overall aroma.

## 1. Introduction

Marselan (*Vitis vinifera* L.) is hybrid cultivar of two famous grape varieties, Cabernet Sauvignon and Grenache [1]. The grape got its name from the French region where it was first bred in 1961. With its strong adaptability to the environment, strong resistance to common diseases, and small berries conducive to extracting aromatic and deep-colored wines with soft tannins, it became a very advantageous grape variety, and was officially registered as a wine grape in France in 1990 [2]. This grape has been gradually accepted and is exclusively used in making some world class wines, especially in “new world” wine countries [3]. Strongly structured and elegant wines are made from Marselan; the result is that it is regarded as a grape variety which is highly adapted to the taste of Chinese consumers [4]. Hence, the planting area has been increasing year by year since Marselan was introduced to China in 2001. At present, China is one of countries with the largest Marselan planting areas. Due to its good performance in China, Marselan might serve as flagship variety in China, like Shiraz for Australian and Sauvignon Blanc for New Zealand [5]. 

Numerous publications have proved the capacity of Marselan for producing high quality wines [6,7], but few studies have dealt with the aroma aspects associated with its high quality. The key aroma compounds of Cabernet Sauvignon and Grenache, from which Marselan was obtained, have been extensively investigated in previous studies [8,9,10]. Several compounds were identified as important, including *β*-damascenone and 3-mercapto-1-hexanol in Grenache wine [8], and *β*-ionone as well as 2-methoxy-3-isobutylpyrazine in Cabernet Sauvignon wine [11]. It is worth noting that the sensory characteristics of the wine from the hybrid are usually different from those of its parents [12,13], as with Marselan wine. It has already been reported that Marselan was shown to produce wines with juicy vegetable and spicy aroma characteristics [14]. A few studies focused on the flavor of Marselan wine through analyzing non-volatile components, and malvidin-3-glucoside was the richest anthocyanin-derived pigment component [1,15]. However, as far as we know, the aromatic and chemical characteristics of Marselan wine have rarely been reported with respect to quantitative data for aroma compounds and their aroma contributions. 

To clarify key aroma compounds, the so-called “sensomics” approach proposed by Schieberle and Hofmann [16] has been successfully used in different kinds of wines [10,17,18,19]. This approach includes aroma extract dilution analysis (AEDA) performed on gas chromatography-olfactometry (GC-O) to discover key odorants, and accurate quantitation followed by aroma reconstitution and omission experiments [20]. In the procedures, the identification and the accurate quantitation of target odor-active regions are crucial [21]. Although the odor-active region can be targeted by GC-O, some key odorants with low thresholds are difficult and time-consuming to identify and quantitate due to the extremely low concentration and the polarity of the compound itself [22]. Comprehensive two-dimensional gas chromatography in combination with time-of-flight mass spectrometry (GC × GC-TOF-MS) has an outstanding performance for identifying compounds [23]. Furthermore GC × GC-TOF-MS, with an excellent ability to separate and enhanced detector capabilities, has been shown to have the advantage in the quantitation of a set of key odorants over foods [24,25]. It can avoid complicated sample pretreatment and simultaneously quantify compounds with large differences of concentration in one run [26]. 

The objective of this research consisted of (1) the identification of the key aroma compounds in Marselan wine by AEDA, (2) the quantitation of key odorants through GC × GC-TOF-MS and calculation of odor active values (OAVs), and (3) validation via aroma reconstitution experiments, sensory description analysis, and omission tests to determine the importance of these compounds.

## 2. Results and Discussion

The volatile compounds of Marselan wine isolated through the solid-phase extraction (SPE) method were evaluated by five assessors among panelists. They confirmed that the extracts exhibited the same typical aroma characteristics compared with the real Marselan wine samples. Thus, the method of SPE was proven to be reasonable for aroma extraction and key aroma compound screening.

### 2.1. Identification of Odor-Active Compounds

AEDA was carried out on the entire set of volatiles isolated by the SPE method. A total set of 43 odorants were detected by GC-O analysis on DB-FFAP and HP-5MS columns in two wine samples (Table 1). The identical odor-active compounds in two different wines of Marselan wines were almost the same. Aroma compounds with high FD factors may make great contributions to the overall aroma of wine [27]. Aroma compounds with FD ≥ 9 were detected as the most odor-active compounds in Marselan wines and the number of these compounds in the 2014 wine was slightly greater than in the 2015 wine. 

*β*-Damascenone, with the greatest FD factors (FD ≥ 2187), was determined as the most important aroma compound in both samples. The aroma characteristics of *β*-damascenone was not the same in different studies, and aromas were found to include cooked apple [28], honey [29], and fruit [30], as well as a honey-like flavor in this study. The second highest FD (FD = 729) was observed for the clove-like eugenol, the butter-like 2,3-butanedione, the rose-like citronellol, the caramel-like furaneol^®^, and the flower-like phenethyl acetate, along with the smoky-like guaiacol and 2-methoxy-4-vinylphenol. Only furaneol^®^ was both detected in the two samples with FD = 729, which was associated with jam or caramel notes. Furthermore, the blueberry-like ethyl isovalerate, the rancid-like 3-methylbutanoic acid, the caramel-like homofuraneol, and cooked-potato odors like methional and methionol were identified with FD ≥ 81 in both samples. The above aroma compounds were suggested as potential contributors to the overall aroma of Marselan red wine considering their high FD factors. Although all the odor-active compounds identified in Marselan wine have been previously reported in other red wines, research has shown that the volatile aroma compositions of wines made from different varieties might only vary in the proportions of those compounds, despite the distinctive aroma perception in varietal wines [31]. The GC-O screening of key odorants was based on their odor threshold in air and not in the wine matrix. In order to further determine their contribution to the overall aroma profile of Marselan wine, the odor activity values (OAVs) of the key odorant needs to be investigated through accurately quantifying the concentration of key odorants to calculate the ratio of the concentration of the odorant and its odor threshold in corresponding matrix [20]. 

### 2.2. Quantitation of Aroma Compounds

**Figure 1:**
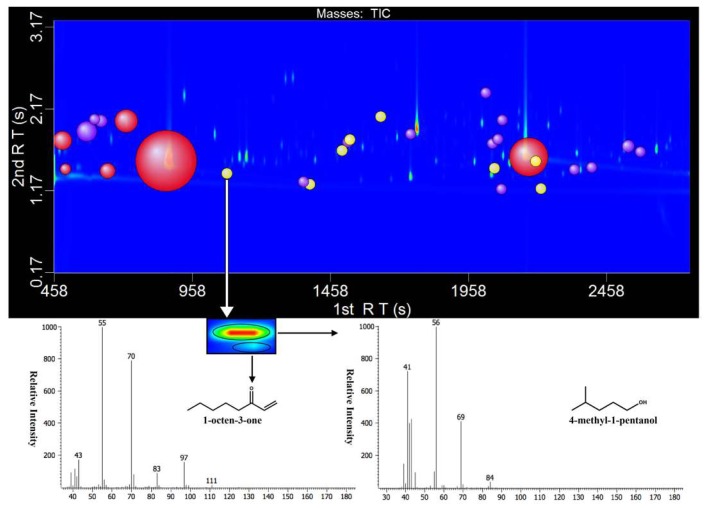
The quantitative compounds in a wine sample displayed using a two-dimensional gas chromatography and time-of-flight mass spectrometry (GC × GC-TOF-MS) contour plot. The size of the bubble represented the relative intensity of the compound’s quantitative ion response, and the minimum size of the bubble was set for clarity. The quantitative compounds were marked by bubbles and the bubbles with different colors represented different concentration ranges. Red bubbles: greater than 1000 μg/L, purple bubbles: between 10 and 1000 μg/L, yellow bubbles: less than 10 μg/L.

The 2014 Marselan wine was selected to be quantified for its composition of more odorants with FD ≥ 9 and higher FD factors. A total of 31 aroma compounds with FD ≥ 9 were quantified by GC × GC-TOF-MS analysis (Table 2 and Table 3). As GC × GC-TOF-MS obtains a higher peak capacity and has enhanced detector capacity, it not only can accurately quantify alcohol compounds exceeding 100 mg/L, but also ng/L concentration of methoxypyrazine compounds in the meantime (Figure 1). For some aldehyde ketone compounds such as 2,3-butanedione, 1-octen-3-one, because the peak time of these compounds is close to some compounds with higher concentration, the sample is often necessary for derivative processing [29,32]. Owing to the excellent separation capabilities of GC × GC-TOF-MS, the sample did not need to be derivatives, and these compounds could be directly separated (Figure 1).

The compound with the highest concentration was 3-methyl-1-butanol (382,940.8 μg/L), followed by phenethyl alcohol (30,823.2 μg/L) and 2-methyl-1-propanol (10,999.4 μg/L) (Table 2). Apparently the three alcohols are volatile constituents of all alcoholic beverages from yeast metabolism [17]. They are produced when the yeast catabolizes amino acids through the Ehrlich pathway [33]. Via this pathway, the amino acids are completely consumed during the early yeast growth phase, resulting in production of the corresponding alcohols later during the yeast stationary phase [34]. There were 14 compounds above 100 μg/L, while eight compounds were found below 10 μg/L. In particular, *β*-ionone and 3-isobutyl-2-methoxypyrazine were at extremely low concentrations (below 1 μg/L).

The OAVs were calculated to evaluate the contributions of odorants. Among these 31 quantified odorants, a total of 21 odorants were verified as important odorants in Marselan wine due to their OAVs above 1.0 (Table 2). *β*-damascenone was not only the odorant with the highest OAV, but also had the greatest FD factors as mentioned before. *β*-damascenone is derived from carotenoid-derived metabolites of grape fruit and also plays an important role in the characteristic aroma in many different varieties of wines such as Cabernet Sauvignon, Merlot, and Shiraz [9,18]. The OAVs of ethyl 2-methylpropanoate, ethyl 2-methylpropanoate, ethyl butanoate, ethyl isovalerate, and isoamyl acetate were all greater than 10. These ester compounds are generally thought to contribute a fruity expression in red wines [35]. Though the concentration of 3-isobutyl-2-methoxypyrazine was only 0.002 μg/L, the OAV value of 3-isobutyl-2-methoxypyrazine reached 10 because of its extremely low threshold (2 ng/L). 3-isobutyl-2-methoxypyrazine is a characteristic compound in Cabernet Sauvignon which imparts sensory characteristics of bell pepper to wines [36]. It is derived from the metabolism and synthesis of grape fruit itself, and is greatly influenced by climatic conditions such as light and water [37]. However, the specific biosynthetic pathway is not clear at present. It is worth noting that the concentration of furaneol^®^ exceeded 100 μg/L, a value much higher than its threshold (5 μg/L). Furaneol^®^ is considered to be the characteristic aroma several hybrid grape varieties and imparts a caramel-like note at a high concentration and a fruity note at a low concentration [38]. In recent years, it has been studied to identify furaneol glucoside from the hybrid grape variety Muscat Bailey, considered to be an important precursor compound of furaneol^®^ [38]. The reason for the higher furaneol^®^ content in Marselan wine might be the accumulation of furaneol glucoside in the grape fruit, which was released during the alcohol fermentation process. Other than this, some compounds were detected with FD factors, but the OAVs of them were below 1, probably due to the threshold, which was significantly different between in the air and in wine matrix.

### 2.3. Aroma Profile Analysis and Aroma Reconstitution

**Figure 2:**
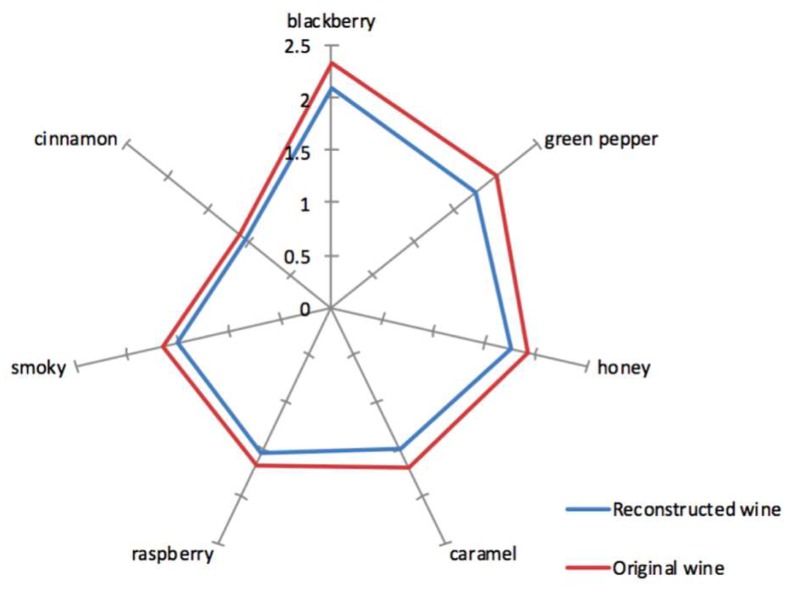
Comparison of the main aroma attribute between Marselan wine and its recombinate wine.

The aroma profile analysis of the Marselan wine revealed that blackberry was the strongest aroma attribute perceivable by panelists, with the highest intensity of 2.3 (red line in Figure 2), followed by moderate green pepper, honey, caramel, raspberry, and smoky aromas. The cinnamon was the weakest perceived aroma attribute.

The global aroma of Marselan wine is a result of natural composition of key odorants in appropriate concentration. An aroma reconstitution experiment was carried out to mimic the characteristic aroma of the Marselan wine and further to confirm the quantitative result. Considering the effect of nonvolatile matrix on aroma perception [49], the reconstitution aroma sample containing 21 key odorants (OAV ≥ 1) was prepared in an odorless wine matrix and was compared with the corresponding real wine. The intensity of seven odor attributes was evaluated. The aroma profile of the recombined wine was similar to that of the original wine, whereas the aroma intensity except cinnamon was slightly lower in the recombined one (Figure 2). However, there is no significant difference in aroma attributes between the Marselan wine and the recombinate wine according to the one-way analysis of variance (ANOVA). Although it is usually suggested that the compounds with low OAVs (less than 1) had no or only little contribution to the overall aroma, there are a lot of perceptual interactions when compounds interact together or interact with the non-volatile components of the wine matrix; these phenomena may affect the perceived intensity of the aroma profile [50]. Therefore the omission test needs to be further implemented to verify the importance of the potential key aroma compounds.

### 2.4. Omission Tests

In order to investigate the significance of the aroma contribution to Marselan wine, a total of 22 omission models were prepared to compare with the reconstitution model by a triangle test. According to Table 3, the data showed that the omission of all esters which were mainly responsible for the fruity in wine were successfully perceived by all panelists with a very high significance (*p* ≤ 0.001). Moreover, the omission of ethyl 2-methylpropanoate as well as ethyl 2-methylbutyrate showed a high significant difference with respect to the reconstitution model (*p* ≤ 0.01) and a significant difference was reflected with the omission of ethyl isovalerate and (*p* ≤ 0.05). Although with the omission of isoamyl acetate and ethyl butanoate a significant difference was detected, the single omission of the two compounds showed no the difference compared with the reconstitution model. This could be the result of the combined action among these compounds. The lack of 3-isobutyl-2-methoxypyrazine was detected with a very significant difference (*p* ≤ 0.001) and the omission of furaneol^®^ (*p* ≤ 0.01) was detected as having a highly significant difference. This result revealed that the green pepper and caramel note could play a very important role in the overall aroma of the sample. 

## 3. Materials and Methods

### 3.1. Wine Samples

The two commercial monovarietal Marselan dry red wines respectively harvested in 2014 and 2015 were selected on the basis of highly representative sensory features through wine expert blind tasting. The wine samples were from the southern foot of Tianshan Mountain in Yanqi County, Xinjiang, China. The basic indicators of two sample wines are as follows: the 2014 Marselan wine (13.5% v/v ethanol, pH 3.50, titratable acidity = 6.6 g/L, total SO_2_ = 65 mg/L), and the 2015 Marselan wine (13.5% v/v ethanol, pH 3.40, titratable acidity = 6.8 g/L, total SO_2_ = 73 mg/L).

### 3.2. Chemicals

All analytical standards used in quantitative analysis and as reference standards during GC-O were purchased from Sigma-Aldrich China Co. (St. Louis, Missouri, USA) with at least 97% purity. These analytical standards were ethyl acetate, ethyl 2-methylbutyrate, 2,3-butanedione, ethyl butyrate, ethyl isovalerat, 1-hexanal, isoamyl acetate, 2-methyl-1-propanol, methyl hexanoate, 3-methyl-1-butanol, ethyl hexanoate, γ-terpinene, 1-octen-3-one, 2,6-dimethylpyrazine, 1-octen-3-ol, 1-heptanol, 3-methylthiopropanal (methional), benzaldehyde, 3-isobutyl-2-methoxypyrazine, linalool, terpinen-4-ol, *β*-cyclocitral, acetophenone, 3-methylbutanoic acid, 3-methylthiopropanol (methionol), citronellol, ethyl laurate, *β*-damascenone, guaiacol, phenethyl alcohol, geraniol, *β*-ionone, 5-butyldihydro-4-methyl-2(3*H*)-furanone (whiskey lactone), 3-hydroxy-2-methyl-4H-pyran-4-one (maltol), 4-hydroxy-2,5-dimethyl-3(2*H*)-furanone (furaneol^®^), γ-nonalactone, 4-hydroxy-5-ethyl-2-methyl-3(2*H*)-furanone (homofuraneol), δ-decalactone, eugenol, and 2-methoxy-4-vinylphenol. Absolute ethanol (≥99.8%, HPLC grade), dichloromethane (≥99.8%, HPLC grade), methanol (≥99.9%, HPLC grade), and 2-octanol (internal standard, IS2) were purchased from Sigma-Aldrich China Co. (St. Louis, MO, USA). Ethyl octanoate-d15 (internal standard, IS1), 2-isobutyl-3-methoxy-d3-pyrazine (internal standard, IS3), 2-phenylethyl acetate-d3 (internal standard, IS4) and 2-methoxy-d3-phenol (internal standard, IS5) were purchased from CDN Isotopes (Quebec, Canada). Ultrapure water was obtained from Milli-Q purification system (Millipore, Bedford, MA).

### 3.3. Isolation of the Volatiles

Volatile compounds were enriched by solid-phase extraction (SPE). [29] Extraction 50 mL of wine sample was at a flow rate of 1 mL/min using a column (LiChrolut EN, Merck, Germany; 500 mg of phase). Before use, the column was rinsed with 6 mL of dichloromethane, 6 mL of methanol and 6 mL of a 12% water–ethanol mixture (ethanol by volume) successively. After the sample was enriched, the column was washed with 20 mL of ultrapure water to eliminate excess pigment, and other low-molecular-weight polar compounds. Then, the sorbent was eluted with 10 mL of dichloromethane and dried with anhydrous sodium sulfate. Finally, under a stream of pure nitrogen, the organic phase was concentrated to a volume of 250 μL and stored at −20 °C before analysis. 

### 3.4. Gas Chromatography-Mass Spectrometric/Olfactometry (GC-MS-O) 

The instruments of an Agilent 6890 gas chromatograph equipped with an Agilent 5975 mass-selective detector (MSD) (Agilent, Palo Alto, CA, USA) and a sniffing port (ODP 2, Gerstel, Germany) were used to analysis. The analytical columns were made up of a DB-FFAP polar column (60 m × 0.25 mm i.d., 0.25-μm film thickness, Agilent, Torrance, CA) and a HP-5MS non-polar column (30 m × 0.25 mm i.d., 0.25-μm film thicknesses, Agilent, Torrance, CA). Helium was used to be as carrier gas at flow rate of 2 mL/min. Aroma extraction of wine sample (1 μL) was injected into the front inlet programmed in splitless mode, and the oven temperature was initially held at 45 °C for 2 min, then raised to 230 °C at 4 °C/min and held for 15 min. The supplemented effluent with helium was split to the olfactory port fixed at the back of the GC detector. The sniffing time was 45 min for each analysis and the capillary, which was connected with the sniffing port, was kept at 250 °C. The data acquisition (electron impact (EI) at 70 eV) was in scan mode with an *m/z* range of 35–400 for compound identification. 

GC-O analysis was conducted by four well-trained assessors (two females and two males) from the Laboratory of Brewing Microbiology and Applied Enzymology at Jiangnan University. The assessors first analyzed the extracts on both DB-FFAP column and HP-5MS column and recorded the retention time and descriptors of the odor peak for each compound. After discussion and checking the aroma descriptor with the chemical standards, a lexicon for GC-O analysis of Marselan wine was generated. After some aroma characteristic remembering and recognition tests, aroma extract dilution analysis (AEDA) was used for searching important odorants.

### 3.5. Aroma Extract Dilution Analysis (AEDA)

AEDA was applied to screen for the most potent odor-active compounds. Aroma extracts were stepwise diluted with dichloromethane (1:2(v/v)) to yield dilutions of 3, 9, 27, etc., finally up to 2187 relative to the initial extracts. Each dilution was analyzed by GC-O on the DB-FFAP column under the same temperature programming conditions until no more odorant appeared. The flavor dilution (FD) factor of each compound was defined as the maximum dilution at which the odorant could be finally perceived by at least three of four assessors. Identification of aroma compounds was achieved by comparing their odors, retention indices (RI) on both columns, and mass spectra with those of pure standards. RI was calculated using a series of standard linear *n*-alkanes (C5–C30) under the same chromatographic conditions. Furthermore, in order to confirm the identification of the odor-active compounds, samples were run on GC × GC-TOF-MS.

GC × GC-TOF-MS The Leco Pegasus 4D GC × GC-TOF-MS hardware system includes Agilent GC model 7890B, the Leco dual nozzle thermal modulator system, and the secondary column thermostat connected to the time of flight mass spectrometer. The column consisted of a one-dimensional chromatographic polar column DB-FFAP (60 m × 0.25 mm × 0.25 μm, Agilent Technologies, Palo Alto, CA, USA) and a two-dimensional chromatographic medium polarity column Rxi-17Sil MS (1.5 m × 0.25 mm × 0.25 μm, Restek, Bellefonte, PA, USA). The front inlet was programmed in splitless mode, the primary oven temperature program conditions were as follows: the initial temperature was 45 °C for 2 min, then raised at 4 °C/min to 230 °C and held for 15 min. The secondary oven temperature was 5 °C higher than the primary oven during the chromatographic run. The modulator temperature was offset +15 °C from primary oven and the modulation time was set at 4 s. The ion source voltage and temperature was 70 eV, 230 °C respectively. The transfer line temperature was 240 °C. The detector voltage was 1430 V. The acquisition mass range was 35–400 amu and the acquisition frequency was 100 spectra/s. The collected data was processed by LECO ChromaTOF workstation, automated peak find and spectral deconvolution with a baseline offset of 0.5.

### 3.6. Quantitative Analysis

The solution of standard compounds was prepared in a model wine (13.5% v/v ethanol solution in Milli-Q-water with 6.6 g/L tartaric acid and adjusted to pH 3.5 with NaOH). Ethyl octanoate-d15 (177.0 μg/L), 2-octanol (91.9 μg/L), 2-isobutyl-3-methoxy-d3-pyrazine (0.198 μg/L), 2-phenylethyl acetate-d3 (210.8 μg/L), and 2-methoxy-d3-phenol (156.4 μg/L) were added to the solutions used as internal standards. The range of compound concentration was listed in Table 4. The SPE method used to extract aroma standard compounds in solution is similar to the above mentioned. The extraction volume for the solution was 25 mL and it was passed through the column at a flow rate of 1 mL/min, but the final sorbent was eluted with 1.5 mL of dichloromethane. For GC × GC-TOF-MS analysis, the extraction (1 μL) was injected into the front inlet in splitless mode. The relative area of each compound (area of compound/area of internal standard) was plotted against the respective compound concentration. Linear regression with at least six concentration levels for each standard compound was constructed by least square linear regression. The standard curve and validation data are shown in Table 4. The LODs (Limit of Detection) were calculated as the analyte concentration of a standard that produced a signal-to-noise ratio of 3 and the LOQs (Limit of Quantitation) were calculated as the analyte concentration of a standard that produced a signal-to-noise ratio of 10. 

### 3.7. Aroma Profile Analysis 

#### 3.7.1. Panel

Thirteen panelists (eight females and five males), between 20 and 25 years old were recruited from the Laboratory of Brewing Microbiology and Applied Enzymology at Jiangnan University for aroma descriptive analysis. They were provided informed consent and came voluntarily and were paid for their participation.

#### 3.7.2. Panel Training and Performance 

Panelists were all trained for at least 6 months on a weekly basis to recognize and describe the odor qualities of a wide range of odorants and products. “Le nez du vin” (Jean Lenoir, Provence, France) was used as the aroma standard to help panelists to recognize and describe the wine odor qualities of 54 odorants. After basic training, the Marselan wine samples were provided to the panelists in the consensus training session for generating a lexicon for Marselan wine. After discussion, a consensus was reached on the main aroma characteristics of the wines, and seven major aroma attributes were selected for descriptive analysis: caramel, cinnamon, smoky, green pepper, honey, raspberry, and blackberry. The standard reference for these attributes were all from the “Le nez du vin” (Jean Lenoir, Provence, France). The vials with different numbers corresponded to their respective aroma attributes in “Le nez du vin” (number 13—raspberry, number 17—blackberry, number 27—honey, number 30—green pepper, number 41—cinnamon, number 51—caramel, and 54—smoky). After training with these standards, the performance of the panel was assessed for each sensory attribute separately by adding these different odors in real Marselan wine samples. It showed that there was a significant difference in the sample effect, and there were no significant differences in the sample–panelist effect and the sample–session effect. That was to say, the panel had good performance in attribute discrimination and the agreement between panelists, as well as repeatability. 

### 3.8. Descriptive Analysis 

With higher FD values than the 2015 wine sample (Table 1), the 2014 wine sample was chosen to be evaluated by aroma descriptive analysis, and then further verified by aroma reconstitution. The wine (15 mL) was poured into a glass vessel (45 mL) and analyzed by scoring the intensity of each attribute on a seven-point scale (steps of 0.5) from 0 (not perceivable) to 3 (strongly perceivable). Samples were analyzed in triplicate, and during the session, the assessors evaluated these samples with a 5 min break after each sample in separated sensory booths according to the standards ISO 8589:2007 [51]. The aroma intensity for each attribute was averaged.

### 3.9. Aroma Reconstitution

The aroma compounds with FD ≥ 9 (Table 2) of wine were added into an odorless wine according to their occurring concentrations. Preparation of the odorless wine was as follows: the wine sample was extracted by the SPE method until the remaining solution was odorless, then through freeze-drying to obtain lyophilizate matrix. The lyophilizate matrix was dissolved by aqueous solutions containing 13.5% of alcohol, and pH was adjusted to 3.5 by tartaric acid before the recombination. The panelists were asked to evaluate the intensity of seven attribute corresponding to the original wine was scored on a seven-point scale (steps of 0.5) from 0 (not perceivable) to 3 (strongly perceivable).

### 3.10. Aroma Omission Tests 

A total of 22 aroma omission models were performed to determine the contribution of certain compounds. Each model was compared with two complete reconstitution by a triangle test and three glass of sample was randomly labeled with numbers. The panelists (six females a four males) were asked to sniff the samples and then select the odd one.

### 3.11. Data Analysis

Quantification data was calculated as mean values with standard deviation from replicate determination (Microsoft Excel 2019, Redmond, WA, USA). The sensory data was analyzed through one-way analysis of variance (ANOVA) with SPSS 24.0 (SPSS Inc., Chicago, IL, USA).

## 4. Conclusions

This study preliminarily analyzed the aroma characteristics and the key odorants of Marselan wine. In addition, the qualitative and quantitative determination of key odorants was verified by a reconstitution experiment. A total of 43 odor-active compounds were detected by GC-O and 31 compounds with FD ≥9 were quantified by GC × GC-TOF-MS. The reconstitution experiment that compared the samples was recombined successfully through 21 aroma compounds with OAV > 1. The omission test revealed that ethyl 2-methylpropanoate, ethyl 2-methylbutyrate, ethyl isovalerate, 2,3-butanedione, 1-octen-3-one, 3-isobutyl-2-methoxypyrazine, *β*-damascenone, guaiacol, *β*-ionone, and furaneol^®^ were important to the overall aroma. However, the non-volatile components of the wine matrix may interact with the aroma compounds; as a result, the volatility of the aroma compounds could be influenced. For further research, it is essential to investigate the interaction of the non-volatile components and aroma compounds on the molecular level to better understand the perception of the aroma in wine.

## Figures and Tables

**Table 1:** Aroma compounds identified in Marselan wine by GC-O.

No.	Compounds ^a^	Odor Description	FD		DB-FFAP		HP-5	
			2014	2015	LRI ^b^	RI	LRI ^c^	RI
1	ethyl acetate	pineapple	3	3	896	907	659	648
2	ethyl 2-methylpropanoate	strawberry	9	27	956	976	760	751
3	2,3-butanedione	butter	729	243	967	985	593	574
4	ethyl butanoate	banana	81	27	1018	1013	744	762
5	ethyl 2-methylbutyrate	apple	27	9	1050	1049	826	842
6	ethyl isovalerate	blueberry	243	81	1068	1063	839	853
7	1-hexanal	grass	3	1	1088	1083	780	
8	2-methyl-1-propanol	solvent	81	9	1099	1086	647	626
9	isoamyl acetate	banana	9	3	1125	1116	849	862
10	1,4-cineole^*^	pine	3	3	1165	1164	1010	
11	methyl hexanoate	fruity	1	3	1190	1193	907	
12	3-methyl-1-butanol	solvent	81	27	1215	1206	722	730
13	ethyl hexanoate	apple	3	1	1238	1231	971	983
14	*γ*-terpinene	pine	3	3	1252	1238	1060	1053
15	1-octen-3-one	mushroom	9	9	1330	1308	912	947
16	2,6-dimethylpyrazine	roasted nut	3	1	1338	1349	847	853
17	1-octen-3-ol	mushroom	3	3	1450	1443	986	976
18	1-heptanol	fatty	27	9	1460	1451	959	971
19	3-methylthiopropanal	baked potato	81	81	1490	1473	885	909
	(methional)							
20	3-isobutyl-2-methoxypyrazine	pepper	27	27	1517	1533	1179	1181
21	benzaldehyde	almond	9	3	1520	1537	960	930
22	linalool	floral	9	9	1550	1542	1070	1102
23	terpinen-4-ol	turpentine	9	81	1612	1603	1164	1162
24	*β*-cyclocitral	green	1	3	1622	1630	1212	1182
25	acetophenone	almond	9		1645	1650	1052	1065
26	3-methylbutanoic acid	rancid	243	243	1666	1664	839	827
27	3-methylthiopropanol	potato	81	81	1715	1720	987	1000
	(methionol)							
28	citronellol	rose	729	243	1767	1764	1212	1221
29	ethyl laurate	leaf	9	3	1848	1849	1597	1578
30	phenethyl acetate	rose	729	729	1856	1826	1229	1183
31	*β*-damascenone	honey	2187	2187	1859	1831	1365	1397
32	guaiacol	smoky	729	243	1875	1873	1119	1090
33	geraniol	geranium	27	27	1893	1843	1330	1306
34	phenethyl alcohol	flowery	81	27	1903	1919	1099	1072
35	*β*-ionone	violet	27	81	1953	1964	1477	1470
36	5-butyldihydro-4-methyl-	coconut	27	27	1968	1987	1299	1310
	2(3*H*)-furanone							
	(whiskey lactone)							
37	3-hydroxy-2-methyl-	caramel	3	9	1998	1985	1088	1077
	4H-pyran-4-one							
	(maltol)							
38	4-hydroxy-2,5-dimethyl-	caramel	729	729	2043	2045	1115	1098
	3(2H)-furanone							
	(furaneol^®^)							
39	γ-nonalactone	prune	3	3	2050	2054	1362	1388
40	4-hydroxy-5-ethyl-2-methyl-	caramel	243	81	2091	2093	1173	1170
	3(2*H*)-furanone							
	(homofuraneol)							
41	δ-decalactone	apricot	3	1	2176	2195	1573	1578
42	eugenol	clove	729	243	2198	2180	1352	1333
43	2-methoxy-4-vinylphenol	smoky	729	243	2213	2216	1298	1300

^a^ The odorants were identified by comparing their retention indices (RIs), mass spectra, and aroma attributes with those of pure standards (except for compounds marked with ‘^*^’) ^b,c^ The RI in literature. FD: flavor dilution.

**Table 2:** Quantitative data odor thresholds and odor activity values (OAVs) of aroma compounds in Marselan wine.

No.	Compound	Thresholds (μg/L) *^a^*	Concentration (μg/L) *^b^*	RSD *^c^*	OAV
1	ethyl 2-methylpropanoate	15.0 [39]	1847.6	5.0	123.2
2	2,3-butanedione	100.0 [39]	1909.2	8.9	19.1
3	ethyl butanoate	20.0 [39]	400.8	2.7	20.0
4	ethyl 2-methylbutyrate	1.0 [39]	186.7	0.8	186.7
5	ethyl isovalerate	3.0 [39]	56.8	2.3	18.9
6	2-methyl-1-propanol	40,000.0 [39]	10,999.4	1.2	<1.0
7	isoamyl acetate	30.0 [39]	1667.2	7.8	55.6
8	3-methyl-1-butanol	30,000.0 [39]	382,940.8	1.2	12.8
9	1-octen-3-one	0.04 [40]	4.2	1.1	105.7
10	1-heptanol	3.0 [41]	20.5	8.4	6.8
11	3-methylthiopropionaldehyde	0.5 [42]	5.1	7.3	10.2
12	3-isobutyl-2-methoxypyrazine	0.002 [43]	0.02	6.7	10.0
13	benzaldehyde	990.0 [44]	77.3	0.4	<1.0
14	linalool	15.0 [39]	4.4	8.3	<1.0
15	terpinen-4-ol	250.0 [45]	3.6	6.6	<1.0
16	acetophenone	200.0 [46]	51.7	0.2	0.3
17	3-methylbutanoic acid	33.0 [45]	465.2	0.2	14.1
18	3-methylthiopropanol	1000.0 [45]	1514.2	4.1	1.5
19	citronellol	40 [47]	4.8	1.2	<1.0
20	ethyl laurate	5900.0 [48]	37.1	0.6	<1.0
21	phenethyl acetate	250.0 [39]	114.7	2.0	<1.0
22	*β*-damascenone	0.05 [39]	40.6	4.5	812
23	guaiacol	9.5 [39]	51.1	8.9	5.4
24	geraniol	30.0 [39]	12.2	6.2	<1.0
25	phenethyl alcohol	10,000.0 [41]	30,823.2	1.6	3.1
26	*β*-ionone	0.1 [45]	0.1	6.4	1.2
27	5-butyldihydro-4-methyl-	67.0 [45]	1.9	3.2	<1.0
	2(3*H*)-furanone (whiskey lactone)				
28	4-hydroxy-2,5-dimethyl-	5.0 [8]	105.9	2.6	21.2
	3(2*H*)-furanone (furaneol^®^)				
29	4-hydroxy-5-ethyl-2-methyl-	125.0 [8]	408.3	8.5	3.3
	3(2*H*)-furanone (homofuraneol)				
30	eugenol	6.0 [45]	17.0	8.9	2.8
31	2-methoxy-4-vinylphenol	1100.0 [39]	125.8	7.0	<1.0

*^a^* Thresholds were taken from the references. In ethanol-water solution except for refs [42,43,45,48,49] in water. *^b^* Average concentration of triplicates. *^c^* RSD, relative standard deviation of the average concentration.

**Table 3:** Omission tests from complete recombination model.

No.	Compound	Correct Number in All	Significance *^a^*
1	all esters	10/10	***
2	ethyl 2-methylpropanoate	8/10	**
3	ethyl butanoate	5/10	
4	ethyl 2-methylbutyrate	8/10	**
5	ethyl isovalerate	7/10	*
6	isoamyl acetate	4/10	
7	ethyl butanoate, isoamyl acetate	7/10	*
8	2,3-butanedione	7/10	*
9	3-methyl-1-butanol	5/10	
10	1-octen-3-one	7/10	*
11	1-heptanol	3/10	
12	3-methylthiopropionaldehyde	5/10	
13	3-isobutyl-2-methoxypyrazine	9/10	***
14	3-methylbutanoic acid	6/10	
15	3-methylthiopropanol	3/10	
16	*β*-damascenone	9/10	***
17	guaiacol	7/10	*
18	phenethyl alcohol		
19	*β*-ionone	7/10	*
20	4-hydroxy-2,5-dimethyl-	8/10	**
	3(2*H*)-furanone (furaneol^®^)		
21	4-hydroxy-5-ethyl-2-methyl-	3/10	
	3(2*H*)-furanone (homofuraneol)		
22	eugenol	4/10	

^a^ “*”, “**”, and “***” indicate significance at *p* ≤ 0.05, 0.01, and 0.001, respectively.

**Table 4:** Validation data for the chemical standards, quantitative ions, and calibrated intervals using GC × GC-TOF-MS.

Compounds	IS	Quantitative	Calibration Curve			Range	LOD	LOQ	Recovery
		Ion (*m/z*)	Slope	Intercept	*R* ^2^	(μg/L)	(μg/L)	(μg/L)	(%)
ethyl 2-methylpropanoate	IS1	71	0.115	−0.002	0.998	139.3–8915.4	0.664	2.213	108.7
2,3-butanedione	IS2	43	0.049	−0.076	0.999	183.7–95,763.8	0.196	0.655	107.6
ethyl butanoate	IS1	116	0.240	0.0854	0.998	44.1–2824.0	0.236	0.788	106.6
ethyl 2-methylbutyrate	IS1	102	0.076	−0.064	0.997	44.4–2651.6	0.051	0.170	117.6
ethyl isovalerate	IS1	88	0.441	−0.075	0.998	16.4–1048.0	0.186	0.619	114.2
2-methyl-1-propanol	IS2	41	0.014	−0.052	0.997	1563.0–50,017.5	2.054	6.847	123.2
isoamyl acetate	IS1	87	0.140	0.347	0.998	32.2–2048.6	0.187	0.625	115.9
3-methyl-1-butanol	IS1	39	0.002	0.552	1.000	486.2–497,752.0	0.173	0.577	113.6
1-octen-3-one	IS1	70	0.641	−0.023	1.000	1.4–90.1	0.065	0.218	85.4
1-heptanol	IS2	56	0.187	0.002	1.000	13.6–874.2	0.044	0.147	81.7
3-methylthiopropionaldehyde	IS1	48	0.151	−0.006	1.000	2.9–191.4	0.133	0.444	103.2
3-isobutyl-2-methoxypyrazine	IS3	124	0.455	0.120	0.999	0.01-0.2	0.001	0.004	89.7
benzaldehyde	IS1	51	0.460	−0.320	0.994	19.6–1252.0	0.060	0.199	83.9
linalool	IS2	121	0.177	0.003	1.000	10.5–334.5	0.322	1.073	115.5
terpinen-4-ol	IS1	71	0.433	−0.007	0.999	1.2–80.7	0.027	0.091	103.9
acetophenone	IS1	77	0.459	−0.225	0.998	9.6–615.6	0.089	0.296	79.9
3-methylbutanoic acid	IS2	60	0.565	−2.408	0.993	85.3–4269.4	0.079	0.264	82.1
3-methylthiopropanol	IS2	106	0.026	0.019	0.999	48.7–3117.8	0.242	0.808	112.8
citronellol	IS2	69	0.130	−0.005	0.998	3.5–222.4	0.154	0.513	96.6
ethyl laurate	IS1	157	0.474	−0.126	1.000	11.2–715.0	0.010	0.033	89.4
phenethyl acetate	IS4	104	0.155	−0.037	0.997	8.8–563.2	0.536	1.787	101.3
*β*-damascenone	IS4	121	0.060	−0.028	1.000	20.7–1386.9	0.428	1.427	104.8
guaiacol	IS5	124	0.080	−0.012	0.999	11.6–744.8	0.030	0.100	118.8
geraniol	IS4	41	0.445	−0.008	0.999	5.9–187.4	0.087	0.290	109.2
phenethyl alcohol	IS4	122	0.020	−1.375	1.000	829.1–849,017.0	0.360	1.200	92.9
*β*-ionone	IS5	177	0.181	−0.001	0.995	1.3–80.9	0.008	0.027	118.1
5-butyldihydro-4-methyl-	IS4	99	0.238	−0.087	0.999	1.4–88.1	0.008	0.026	124.6
2(3*H*)-furanone									
(whiskey lactone)									
4-hydroxy-2,5-dimethyl-	IS4	128	0.003	0.001	0.996	27.6–884.4	0.663	2.209	96.4
3(2*H*)-furanone									
(furaneol^®^)									
4-hydroxy-5-ethyl-2-methyl-	IS4	57	0.012	0.001	0.999	42.5–681.0	1.059	3.529	107.0
3(2*H*)-furanone									
(homofuraneol)									
eugenol	IS5	164	0.155	0.007	0.999	0.9–59.2	0.007	0.022	113.9
2-methoxy-4-vinylphenol	IS5	135	0.024	−0.085	0.997	11.8–755.0	0.133	0.444	89.1

^a^ The internal standard (IS) used to quantitate the compounds: ethyl octanoate-d_15_ ( IS1, *m/z* = 187), 2-octanol ( IS2, *m/z* = 45), 2-isobutyl-3-methoxy-d_3_-pyrazine (IS3, *m/z* = 127 ), 2-phenylethyl acetate-d_3_ (IS4, *m/z* = 127 ), 2-methoxy-d_3_-phenol (IS5, *m/z* = 127).
